# Timing of Achieving 70% of Energy Requirements in Critically Ill Patients: Association with In-Hospital Mortality and Predictors in a Real-World Medical ICU

**DOI:** 10.3390/nu18101545

**Published:** 2026-05-13

**Authors:** Ya-Ling Wu, Chiann-Yi Hsu, Ya-Ling Wang, Chen-Yu Wang

**Affiliations:** 1 Department of Food and Nutrition, Taichung Veterans General Hospital, Taichung 407219, Taiwan; ylw12@vghtc.gov.tw (Y.-L.W.); ylwan@vghtc.gov.tw (Y.-L.W.); 2 Biostatistics Group, Department of Medical Research, Taichung Veterans General Hospital, Taichung 407219, Taiwan; chiann@vghtc.gov.tw; 3 Department of Nutrition, Hungkuang University, Taichung 433304, Taiwan; 4 Department of Critical Care Medicine, Taichung Veterans General Hospital, Taichung 407219, Taiwan; 5 Department of Nursing, Hungkuang University, Taichung 433304, Taiwan

**Keywords:** critical illness, intensive care unit, caloric target achievement, timing of nutrition support, in-hospital mortality

## Abstract

**Background/Objectives:** The European Society for Clinical Nutrition and Metabolism recommends avoiding full feeding during the first 48–72 h of critical illness and gradually achieving 70–100% of energy requirements within the first week. However, the clinical significance of achieving approximately 70% of estimated energy requirements by day 7 in the intensive care unit (ICU) remains unclear in routine practice. This study investigated whether achieving ≥70% of estimated energy requirements by day 7 was associated with in-hospital mortality and explored clinical factors associated with achieving this target. **Methods:** This retrospective study included critically ill patients who remained in the ICU through day 7 and had complete nutritional data for the first 7 ICU days. Cox proportional hazards regression was performed in a day-7 landmark cohort to investigate the association between day-7 energy adequacy and subsequent in-hospital mortality. Logistic regression analysis was performed to identify the factors associated with achieving ≥70% of estimated energy requirements by day 7. **Results:** Among 507 patients in the day-7 landmark cohort, 355 achieved ≥70% of estimated energy requirements by ICU day 7, and 152 did not. Achievement of the day-7 target was associated with lower in-hospital mortality (adjusted hazard ratio [aHR] 0.50, 95% CI 0.30–0.84; *p* = 0.008), whereas achievement of the same target by day 3 was not significantly associated with in-hospital mortality. Older age and elevated serum albumin levels were independently associated with achieving the day-7 target. In exploratory subgroup analyses, the point estimates were directionally similar across clinically relevant subgroups, but no statistically significant interaction was observed. **Conclusions:** Achievement of ≥70% of estimated energy requirements by ICU day 7 was associated with lower in-hospital mortality, whereas achievement of the same target by day 3 was not significantly associated with in-hospital mortality. These findings should be interpreted as observational and hypothesis-generating.

## 1. Introduction

Critically ill patients frequently experience metabolic stress, systemic inflammation, and gastrointestinal dysfunction during the acute phase of illness. These conditions predispose them to cumulative energy deficits early in the intensive care unit (ICU) stay. Current international guidelines, including those issued by the European Society for Clinical Nutrition and Metabolism (ESPEN), recommend early initiation of enteral nutrition, followed by gradual advancement of caloric delivery over the first week rather than full caloric replacement in the very early phase of critical illness [1,2]. This phase-adapted approach reflects limited metabolic tolerance to exogenous nutrition during the acute stress response.

In routine clinical practice, the progression of energy delivery varies across patients. Hemodynamic instability, feeding intolerance, procedural interruptions, and differences in institutional nutrition protocols lead to gradual and inconsistent advancement of caloric intake. Many ICUs adopt a pragmatic target of achieving approximately 70% of estimated energy requirements by day 7; however, the optimal timing for reaching this level of energy adequacy remains insufficiently defined in real-world settings [3].

Emerging evidence suggests that the timing of adequate nutritional delivery during specific phases of critical illness may be more clinically relevant than early full caloric intake. A 2024 prospective cohort study further showed that adequate nutrition during the later acute phase (days 5–10) was associated with a reduced risk of short-term mortality [4]. The literature on critical care nutrition supports a phase-adapted nutritional strategy. This approach highlights that metabolic tolerance to exogenous energy is limited during the early stress phase and supports gradual increases in caloric delivery over time [5].

More recent observational studies have examined how the timing of energy adequacy relates to clinical outcomes. Yue et al. reported that achieving ≥70% of estimated energy requirements between ICU days 4 and 7 was associated with reduced mortality in a general ICU cohort [6]. Similarly, Wen et al. observed that caloric adequacy during days 3–7 was more strongly associated with favorable outcomes in patients with sepsis than was caloric intake during the first 1 to 2 days [7]. These studies provide insight into the relationship between energy intake and outcomes; however, they primarily examined the association between energy intake and clinical outcomes. The real-world feasibility of achieving predefined energy targets and the clinical factors associated with successful target attainment remain unclear.

From a pathophysiological perspective, gradual increases in caloric delivery during the acute phase are biologically plausible. Persistent endogenous glucose production and the adaptive role of autophagy under stress support this approach [8]. Clinical factors such as disease severity, nutritional status, and gastrointestinal tolerance may influence the ability to achieve energy targets during the first week; however, relevant real-world evidence from ICU cohorts remains limited.

Prior studies have mainly examined associations between caloric adequacy and clinical outcomes. In contrast, less is known about the real-world attainability of a pragmatic day-7 energy target and the clinical factors associated with successful target attainment in routine ICU care. Therefore, this study investigated whether achieving ≥70% of estimated energy requirements by ICU day 7 was associated with in-hospital mortality and explored the clinical factors associated with achieving this target in a real-world medical ICU cohort.

## 2. Materials and Methods

### 2.1. Study Design and Population

This single-center retrospective observational study was conducted in the medical ICU of Taichung Veterans General Hospital. We screened all critically ill adults (aged ≥18 years) admitted between January 2021 and December 2022 who required respiratory support and received enteral nutrition, parenteral nutrition, or both. Patients were eligible if they remained in the ICU through day 7 and had complete nutritional data for the first 7 ICU days. The exclusion criteria were as follows: (1) having a terminal disease at ICU admission, defined as terminal malignancy, progressive and irreversible organ failure precluding definitive therapy, or receipt of palliative or comfort-oriented care; and (2) having a body mass index (BMI) of <18.5 kg/m^2^. The BMI criterion was applied to reduce heterogeneity in early caloric prescription. In our institution, underweight patients (BMI < 18.5 kg/m^2^) are often managed with a more conservative caloric initiation and advancement strategy during the early phase of ICU care because of concern regarding refeeding risk and feeding tolerance. Because refeeding-related risk is clinically relevant in patients with low BMI, yet not uniformly operationalized in routine practice, inclusion of this subgroup could have introduced systematic bias in day-7 target attainment related to intentional clinical restriction rather than comparable nutritional progression across the cohort.

**Figure 1:**
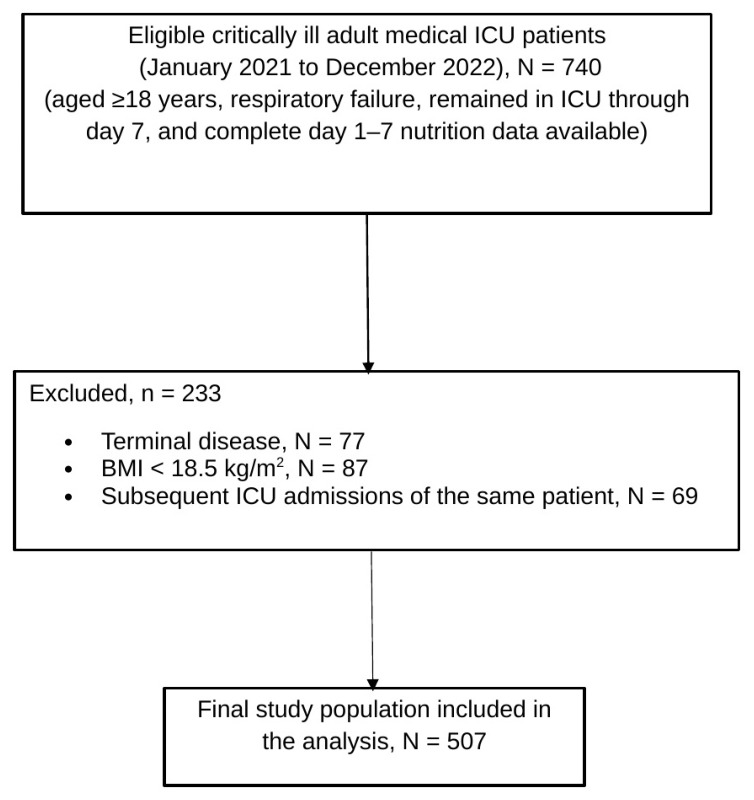
Flowchart depicting patient selection**.** A total of 507 patients who remained in the ICU through day 7 and had complete nutrition data for the first 7 ICU days were included in the final analysis.

Of 740 eligible ICU admissions, 233 were excluded (77 in patients with terminal disease, 87 in patients with a BMI of <18.5 kg/m^2^, and 69 representing subsequent ICU admissions of the same patient). The final study population included 507 patients (Figure 1).

### 2.2. Assessment and Calculation of Nutritional Support

Although indirect calorimetry is often considered a reference method for estimating energy expenditure in critically ill patients [9], it was not routinely available in our ICU during the study period. Therefore, energy requirements were prescribed using a fixed weight-based approach of approximately 25 kcal/kg/day, in accordance with routine ICU practice and guideline-based dietitian assessment [2]. For patients with obesity (BMI ≥ 25 kg/m^2^), adjusted body weight was calculated as follows: adjusted body weight = ideal body weight + 0.25 × (actual body weight − ideal body weight) [10]. Protein requirements were set at 1.0–1.3 g/kg/day for patients without obesity and 1.3 g/kg adjusted body weight/day for those with obesity [2,10].

For the calculation of energy adequacy, total caloric intake included calories derived from enteral nutrition and intravenous caloric exposure. To further characterize nutritional administration, the enteral nutrition ratio was calculated descriptively as the proportion of calories derived from enteral nutrition relative to total caloric intake during the observation period. Intravenous caloric exposure comprised calories from prescribed parenteral nutrition as well as documented non-nutritional intravenous caloric sources, such as propofol, dextrose-containing intravenous fluids, and citrate. Protein intake was calculated from enteral and parenteral nutrition only. Nutritional adequacy was calculated as follows: energy intake (%) = (actual total caloric intake/estimated energy requirement) × 100%; protein intake (%) = (actual protein intake/estimated protein requirement) × 100%; and protein intake (g/kg body weight) = actual protein intake (g)/body weight (kg). Because formal parenteral nutrition could not be reliably separated from non-nutritional intravenous caloric sources in the retrospective dataset, route-related analyses were based on enteral nutrition ratio and intravenous caloric exposure rather than a strict EN/PN/mixed support classification.

### 2.3. Data Collection

The following data were collected from electronic medical records: demographic characteristics; comorbidities, assessed using the Charlson Comorbidity Index (CCI); disease severity, assessed using the Acute Physiology and Chronic Health Evaluation II (APACHE II) score and the Sequential Organ Failure Assessment (SOFA) score; nutritional risk, assessed using the modified Nutrition Risk in the Critically Ill (mNUTRIC) score; laboratory parameters (albumin, C-reactive protein, and hemoglobin); ICU length of stay; mechanical ventilation data; and clinical outcomes.

### 2.4. Severity, Nutritional Risk, and Comorbidity Assessment

Illness severity, organ dysfunction, nutritional risk, and comorbidity burden were assessed at ICU admission using APACHE II, SOFA, mNUTRIC, and CCI, respectively. In the primary regression analyses, these variables were considered mainly as continuous variables. For descriptive stratification, cutoffs reported in prior literature were used to define relatively higher-risk groups: APACHE II ≥ 25, SOFA > 8, mNUTRIC ≥ 5, and CCI > 4 [11,12,13,14,15,16,17,18,19].

The study protocol was approved by the Institutional Review Board of Taichung Veterans General Hospital (approval number: CE23179A). The requirement for written informed consent was waived because of the retrospective nature of this study and the use of anonymized data. This study adhered to the ethical principles of the Declaration of Helsinki.

### 2.5. Statistical Analysis

Continuous variables were compared using the Mann–Whitney U or Kruskal–Wallis test and are presented as medians (interquartile ranges). Categorical variables were compared using the χ^2^ test or Fisher’s exact test and are presented as numbers (%). Cox proportional hazards regression was performed to identify the association between achieving ≥70% of the energy target and in-hospital mortality. Logistic regression analysis was performed to identify clinical factors associated with achieving ≥70% of the energy target by ICU day 7. Survival was compared between the ≥70% and <70% energy target groups using Kaplan–Meier curves and the log-rank test. Missing data were minimal. Only baseline serum albumin had missing values (10/507, 2.0%). Because missingness was minimal, these values were imputed using the series mean. A two-sided *p* value < 0.05 was considered statistically significant. All analyses were performed using SPSS version 22.0 (IBM Corp., Armonk, NY, USA). Because exposure status was defined according to nutritional intake accrued through ICU day 7, survival analyses were performed using a day-7 landmark design, with follow-up beginning on ICU day 7 among patients who remained in the ICU and had complete nutritional data through that time point. Variables for multivariable models were selected on the basis of clinical relevance and potential confounding rather than univariable significance alone, with attention to overlap and collinearity among severity-related variables. Therefore, APACHE II and SOFA were not entered simultaneously into the same primary model.

## 3. Results

### 3.1. Baseline Characteristics and Clinical Outcomes of Hospital Survivors and Nonsurvivors

Among the 507 patients included in this retrospective study, 65 (12.8%) died during hospitalization, and 442 (87.2%) survived. The nonsurvivors were significantly older than survivors, with a higher median age [72 (64–82) vs. 69 (56–79) years, *p* = 0.015; Table 1].

The nonsurvivors had greater disease severity than did the survivors, as indicated by higher median APACHE II scores (*p* = 0.013) and SOFA scores (*p* = 0.022). Furthermore, the nonsurvivors also had significantly lower serum albumin levels than did the survivors [median 2.6 (2.3–2.9) vs. 2.9 (2.5–3.3) g/dL, *p* < 0.001] and higher C-reactive protein levels (*p* = 0.010).

The proportion of individuals with high nutritional risk, defined as an mNUTRIC score of ≥5, was higher in the nonsurvivors group than in the survivor group (96.9% vs. 89.1%, *p* = 0.049). By contrast, the proportion of individuals achieving ≥70% of the target energy intake by ICU day 7 was significantly higher in the survivor group than in the nonsurvivor group (71.9% vs. 56.9%, *p* = 0.014). Route-related nutritional variables were also summarized descriptively. Survivors had a higher EN ratio than nonsurvivors, whereas the proportions of patients receiving EN only and intravenous caloric exposure only were low in both groups (Table 1).

### 3.2. Association Between Day-7 Energy Adequacy and In-Hospital Mortality

**Figure 2:**
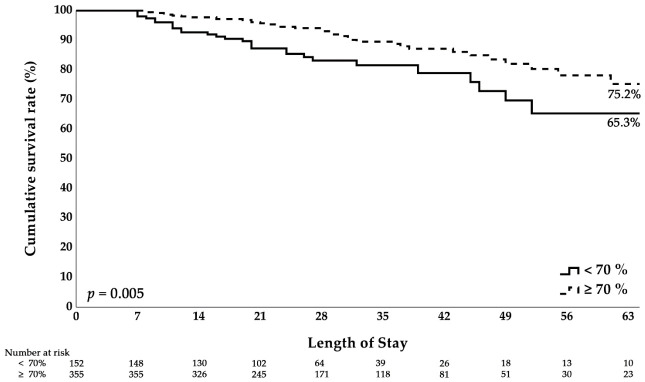
Kaplan–Meier curves for subsequent in-hospital mortality from the ICU day-7 landmark according to day-7 energy adequacy.

Kaplan–Meier survival analysis (Figure 2) revealed that patients who achieved ≥70% of the target energy intake by ICU day 7 had significantly higher cumulative in-hospital survival than those who did not achieve this target (*p* = 0.005).

Survival probability stratified by achieving ≥70% of estimated energy requirements on day 7. Patients meeting the day-7 target demonstrated significantly better survival than did those not meeting this target. Statistical comparison between curves was performed using the log-rank test. *p* < 0.05 indicated statistical significance.

In the multivariable Cox proportional hazards model (Table 2), after adjustment for age, sex, SOFA score, and serum albumin level, achieving ≥70% of the target energy intake by ICU day 7 was independently associated with reduced in-hospital mortality (hazard ratio [HR] = 0.50, 95% confidence interval [CI]: 0.30–0.84, *p* = 0.008). Lower serum albumin levels were also independently associated with higher in-hospital mortality (HR = 0.49, 95% CI: 0.30–0.78, *p* = 0.002). By contrast, achievement of ≥70% of estimated energy requirements by ICU day 3 was not significantly associated with in-hospital mortality (univariable HR = 0.71, 95% CI: 0.43–1.16; *p* = 0.171). Sensitivity analyses excluding patients who died on ICU day 7 or within the first 8 or 9 ICU days yielded broadly similar results (Tables S1–S3 and Figure S1).

### 3.3. Clinical Characteristics of the Achievement and Nonachievement Groups

Patients were stratified into an achievement group (*n* = 355) and a nonachievement group (*n* = 152) on the basis of whether they achieved ≥70% of the target energy intake by ICU day 7. As shown in Table 3, the nonachievement group was significantly younger than the achievement group (*p* = 0.024). However, the nonachievement group had greater disease severity than did the achievement group, as indicated by higher APACHE II scores (*p* = 0.045) and higher SOFA scores (*p* = 0.009). The nonachievement group also had significantly lower serum albumin levels than did the achievement group [2.7 (2.3–3.1) vs. 2.9 (2.5–3.3) g/dL, *p* = 0.002]. Route-related nutritional variables were also compared descriptively between the achievement and nonachievement groups. The achievement group had a higher EN ratio, whereas EN-only and intravenous-caloric-exposure-only categories were infrequent overall (Table 3).

### 3.4. Predictors of Achieving ≥70% of Target Energy Intake by ICU Day 7

Logistic regression analysis was performed to identify factors associated with achieving ≥70% of the target energy intake by ICU day 7 (Table 4). In the multivariable model, two factors were independently associated with achieving this target. Older age was modestly associated with a higher likelihood of achieving the target. Furthermore, a higher serum albumin level was significantly associated with a higher likelihood of achieving the target (OR = 1.61, 95% CI: 1.14–2.29, *p* = 0.007).

### 3.5. Results of Subgroup Analyses

**Figure 3:**
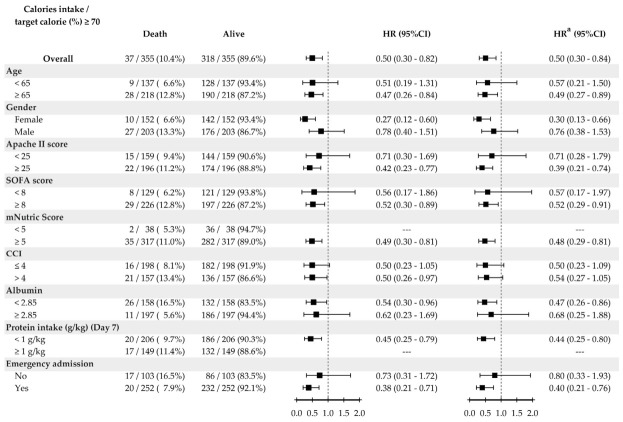
Subgroup analysis of the association between day 7 energy adequacy and in-hospital mortality (adjusted Cox model). Squares indicate HR estimates, and horizontal lines indicate 95% CIs.

The results of subgroup analyses are presented in Figure 3. Achieving ≥70% of estimated energy requirements by ICU day 7 was associated with reduced in-hospital mortality in the adjusted model (adjusted HR = 0.50, 95% CI: 0.30–0.84). In higher-risk subgroups, including patients with hypoalbuminemia, older patients, patients with high nutritional risk, and patients with insufficient protein intake by day 7, the point estimates also favored achieving ≥70% of energy requirements by day 7. The point estimates were directionally similar across clinically relevant subgroups, and no statistically significant interaction was observed.

Hazard ratios (HRs) for in-hospital mortality are shown. The main variable of interest was day-7 energy adequacy (≥70%). Multivariable Cox proportional hazards regression models were adjusted according to the covariates included in the primary multivariable model shown in Table 2. Day-7 energy adequacy remained a protective factor after adjustment. *p* < 0.05 indicated statistical significance.

## 4. Discussion

In our retrospective cohort of critically ill patients, achieving ≥70% of estimated energy requirements by ICU day 7 was associated with reduced in-hospital mortality. By contrast, achievement of the same target by ICU day 3 was not significantly associated with in-hospital mortality in the present analyses. The novelty of the present study lies not only in examining the outcome association of day-7 energy adequacy but also in evaluating the practical attainability of this target and the patient characteristics associated with successful attainment in routine ICU practice.

These findings suggest that, within this day-7 landmark cohort, nutritional adequacy achieved later in the first week may be more informative than very early attainment of the same target. This interpretation is consistent with phase-adapted nutritional therapy and with prior observational studies reporting that energy adequacy during the later acute phase is more closely associated with favorable outcomes than intake during the first 1–2 days of critical illness [3,4,6,7]. However, because these findings were derived from an observational landmark analysis, they should be interpreted as associations rather than evidence of a causal timing effect.

These exploratory subgroup findings should be interpreted cautiously. Although the point estimates were directionally similar across clinically relevant subgroups, no statistically significant interaction was detected; therefore, these analyses should not be interpreted as demonstrating uniform effects across all subgroups.

From a physiological perspective, metabolic and inflammatory responses during the early acute phase of critical illness remain unstable and may limit tolerance to rapid increases in exogenous energy delivery. Impaired substrate utilization, feeding intolerance, and ongoing inflammatory stress may reduce the benefit of early full caloric provision, as reported in prior studies [20,21].

Our observations relate to an unresolved problem noted in the ESPEN guidelines. Although gradual advancement of energy provision during the first week is recommended, the optimal timing for achieving approximately 70% of energy requirements remains unclear [1,2]. The present study provides a real-world clinical context to this question; however, it cannot offer definitive guidance because of the observational design.

### 4.1. Optimal Timing for Achieving Energy Targets

The observed association between achieving energy targets by ICU day 7 and reduced mortality may reflect the clinical feasibility of gradually increasing caloric delivery during the early phase of critical illness rather than rapidly increasing intake within the first few days after ICU admission. This interpretation is consistent with recent observational evidence indicating more favorable outcomes with progressive energy provision than with early aggressive energy provision [22]. Yue et al. reported that mortality was the lowest among patients who achieved approximately 70% of energy requirements between ICU days 4 and 7 [6], whereas Wen et al. reported that caloric adequacy during ICU days 3–7 was more strongly associated with favorable outcomes in patients with sepsis than was caloric intake during the first 1 to 2 days [7]. These findings suggest that the timing of achieving energy adequacy during the first week is more relevant to clinical outcomes than is very early caloric attainment. However, because the studies, including the present study, were observational in nature, the observed associations should be interpreted as hypothesis-generating rather than prescriptive for clinical practice.

### 4.2. Physiological Rationale for Gradual Feeding

Several pathophysiological mechanisms may explain why early aggressive feeding does not provide additional clinical benefit during the acute phase of critical illness. Under severe stress, endogenous glucose production remains elevated, and autophagy plays a crucial role in cellular repair and metabolic adaptation. Excessive exogenous energy provision during this period may interfere with these adaptive processes and increase metabolic burden [8].

Early nutritional support that exceeds actual energy expenditure may increase the risk of overfeeding. This can lead to high carbon dioxide production and increased metabolic stress, which may be poorly tolerated in patients who are not yet physiologically stable. The mismatch between energy provision and energy expenditure has been associated with prolonged mechanical ventilation and metabolic complications in critically ill patients [22].

Evidence from randomized trials, including the study by Arabi et al. [23], indicates that early full feeding does not reduce mortality compared with more conservative caloric strategies. In this context, the absence of an additional survival benefit from achieving energy targets as early as ICU day 3 in the present cohort appears physiologically plausible and is consistent with the findings reported by Arabi et al. Direct comparisons should still be interpreted cautiously because of differences in study design and patient populations.

Current guidelines generally favor early enteral nutrition when feasible, whereas supplemental intravenous or parenteral caloric support may be used when enteral delivery is insufficient or poorly tolerated. In real-world ICU practice, achievement of energy targets often reflects not only prescribed caloric goals but also dynamic changes in tolerance, interruptions, and the need for supplemental parenteral nutrition. Thus, the route of delivery should be interpreted as part of an individualized nutrition strategy rather than as a simple binary exposure. This perspective is also consistent with recent reviews emphasizing more personalized EN/PN integration in critically ill patients [2,3,24]. In supplementary analyses, adjustment for the EN ratio did not materially alter the association between day-7 target achievement and in-hospital mortality (Table S4). This finding provides supportive evidence that the observed association was not fully explained by the relative contribution of enteral calories alone.

### 4.3. Clinical Predictors of Achieving ≥70% of Energy Targets by ICU Day 7

We identified the clinical factors associated with achieving ≥70% of estimated energy requirements by ICU day 7. This study provides a complementary perspective to recent investigations that focused primarily on the association between the timing of energy adequacy and clinical outcomes [4,6,7]. However, the clinical factors associated with successful achievement of predefined energy targets in routine practice remain unclear.

Our analysis revealed that older age and higher serum albumin levels were independently associated with a higher likelihood of achieving ≥70% of estimated energy requirements by ICU day 7. Serum albumin may reflect both nutritional reserve and underlying inflammatory status, which may affect tolerance to nutritional advancement in critically ill patients [25,26]. Illness severity-related variables were included in the statistical model; however, they were not independently associated with achieving the target after multivariable adjustment.

Few studies have examined patient characteristics associated with achieving energy targets during the first week of critical illness. We analyzed these factors while accounting for disease severity and nutritional risk to better characterize clinical profiles associated with successful energy delivery. This analysis complements prior studies that focused mainly on associations between caloric adequacy and clinical outcomes rather than on predictors of target attainment [27,28].

From a clinical perspective, our findings suggest that patients with lower serum albumin levels are more likely to fall short of planned energy delivery during the first week of ICU stay. These findings may help dietitians identify patients who require closer monitoring of nutritional progression or earlier adjustment of feeding strategies. However, because this study was retrospective in nature, the observed associations should be interpreted cautiously and considered hypothesis-generating.

### 4.4. Strengths and Limitations

This study has several strengths in the context of routine critical care. First, the analysis was performed using daily nutritional records collected during ICU care, which enabled us to comprehensively assess energy delivery over the first week rather than relying on a single summary measure. This approach provides a practical view of how nutritional targets are achieved under real-world bedside conditions, where interruptions, hemodynamic instability, and tolerance-related problems are common. Second, this study focused on achieving ≥70% of estimated energy requirements by ICU day 7, a clinically relevant target that aligns with progressive energy advancement during the acute phase of critical illness. Finally, we not only analyzed the association between day-7 energy adequacy and mortality but also identified the clinical characteristics associated with achieving this target. The findings revealed that older age and higher serum albumin levels were independently associated with achieving the day-7 target, providing additional practice-oriented insight beyond outcome associations alone. Overall, our findings provide real-world observational evidence suggesting the potential clinical relevance of energy delivery trajectories during the first week of ICU stay. The findings may help clinicians interpret day-7 energy adequacy as a marker of nutritional delivery in practice.

This study has several limitations. First, the retrospective single-center design precludes causal inference, although it allowed for the collection of detailed longitudinal nutritional data that are often difficult to obtain in larger multicenter datasets. Second, energy requirements were prescribed using a fixed weight-based approach rather than indirect calorimetry. This may have led to underestimation or overestimation of true energy requirements in individual patients and may not fully capture metabolic variability during critical illness; however, this approach remains common in routine ICU practice, where indirect calorimetry is not always available. Third, most patients were treated in a medical ICU; therefore, caution is required when extrapolating these findings to surgical or other specialized ICU populations.

Importantly, the study included only patients who remained in the ICU through day 7 and had complete nutritional data for the first 7 ICU days. Although the day-7 landmark design reduced misclassification of time-dependent nutritional exposure, it may have introduced survivor-related selection bias. Reverse causality also cannot be excluded, because patients who were clinically more stable, had better gastrointestinal tolerance, or experienced fewer interruptions to feeding may have been more likely to achieve the day-7 energy target. Although multivariable models were adjusted for clinically relevant covariates, residual confounding remains possible. Feeding tolerance, gastrointestinal intolerance, interruptions to feeding, and clinician-driven restrictions in nutritional advancement were not systematically recorded and therefore could not be directly analyzed.

Route-specific analyses were also limited. Formal parenteral nutrition could not be fully separated from non-nutritional intravenous caloric sources in the retrospective dataset; therefore, route-related analyses were based on enteral nutrition ratio and intravenous caloric exposure rather than a strict EN/PN/mixed support classification. In addition, the number of patients receiving EN only or intravenous caloric exposure only was small, limiting the stability and interpretability of route-specific multivariable analyses. Finally, exclusion of patients with BMI <18.5 kg/m^2^ may limit generalizability, particularly to underweight and older ICU populations. This criterion was prespecified because low-BMI patients in our institution were often managed with more cautious early caloric advancement owing to concern for refeeding-related risk, which could have introduced additional heterogeneity in target attainment.

## 5. Conclusions

In this real-world retrospective ICU cohort, achieving ≥70% of estimated energy requirements by day 7 was associated with reduced in-hospital mortality. Achievement of the same target by day 3 was not significantly associated with in-hospital mortality in the present analyses. These findings should be interpreted cautiously, and prospective studies are needed to confirm the optimal timing of energy adequacy.

## Figures and Tables

**Table 1:** Baseline demographic and clinical characteristics of survivors and nonsurvivors during hospitalization.

	Hospital Mortality		*p* Value	
	Alive (*n* = 442)	Death (*n* = 65)		
Age, median (IQR)	69 (56–79)	72 (64–82)	0.015 *	
Male, *n* (%)	259 (58.6%)	40 (61.5%)	0.653	
Body weight (kg), median (IQR)	63.5 (56.0–73.0)	61.0 (53.7–70.5)	0.253	
BMI (kg/m^2^), median (IQR)	24.2 (21.9–27.4)	23.3 (20.4–27.3)	0.222	
APACHE II score, median (IQR)	26 (21–30)	28 (24–32)	0.013 *	
SOFA score, median (IQR)	9 (6–12)	10 (8–13)	0.022 *	
CCI, median (IQR)	4 (2–6)	5 (2–7)	0.051	
mNUTRIC score, median (IQR)	7 (6–8)	7 (6–8)	0.004 **	
mNUTRIC score ≥ 5, *n* (%)	394 (89.1%)	63 (96.9%)	0.049 *	
Ventilator day, median (IQR)	10 (8–16)	12 (9–20)	0.001 **	
Days of ICU stay, median (IQR)	13 (9–20)	14 (10–21)	0.612	
Days of hospital stay, median (IQR)	28 (20–40)	22 (12–34)	0.001 **	
Emergency admission, *n* (%)	313 (70.8%)	40 (61.5%)	0.129	
Laboratory data, median (IQR)				
Hgb (g/dL)	9.7 (8.3–12.0)	9.2 (8.2–10.7)	0.084	
CRP (mg/dL)	6.9 (1.7–16.5)	12.9 (5.5–18.6)	0.010 *	
Albumin (g/dL)	2.9 (2.5–3.3)	2.6 (2.3–2.9)	<0.001 **	
EN ratio, median (IQR)	93.4 (80.7–98.1)	84.6 (70.5–93.4)	<0.001 **	
EN only, *n* (%)	37 (8.4%)	3 (4.6%)	0.294	
IV caloric exposure only, *n* (%)	2 (0.5%)	0 (0%)	1.000	
Day 3, median (IQR)				
Calorie intake (kcal)	1107 (764–1401)	956 (614–1243)	0.038 *	
Calorie intake/target calorie (%) ≥ 70, *n* (%)	238 (53.8%)	29 (44.6%)	0.164	
Calorie intake/target calorie (%)	75.3 (50.4–96.5)	64.4 (44.3–92.2)	0.113	
Calorie intake (kcal/kg)	16.9 (11.3–22.8)	15.3 (8.8–21.9)	0.157	
Protein intake (g/kg)	0.7 (0.4–0.9)	0.6 (0.3–0.9)	0.169	
Day 7, median (IQR)				
Calorie intake (kcal)	1302 (985–1541)	1200 (596–1513)	0.096	
Calorie intake/target calorie (%) ≥ 70, *n* (%)	318 (71.9%)	37 (56.9%)	0.014 *	
Calorie intake/target calorie (%)	87.2 (68.3–105.8)	78.7 (46.9–105.2)	0.147	
Calorie intake (kcal/kg)	19.8 (15.2–25.2)	18.7 (11.3–24.8)	0.162	
Protein intake (g/kg)	0.8 (0.6–1.0)	0.7 (0.3–1.0)	0.037 *	

Data are presented as median interquartile range (IQR) or *n* (%). Comparisons between survivors and nonsurvivors were performed using the Mann–Whitney U test for continuous variables, the chi-square test for categorical variables, and Fisher’s exact test when appropriate. * *p* < 0.05 and ** *p* < 0.01 indicate statistical significance.

**Table 2:** Predictors of hospital mortality.

	Univariable Model		Multivariable Model	
	HR (95%CI)	*p* Value	HR (95%CI)	*p* Value
Calorie intake/target calorie ≥ 70 (%)				
Day 3	0.71 (0.43–1.16)	0.171		
Day 7	0.50 (0.30–0.82)	0.006 **	0.50 (0.30–0.84)	0.008 **
Age, per year increment	1.02 (1.00–1.03)	0.073	1.02 (1.00–1.04)	0.027 *
Male vs. Female	1.12 (0.68–1.85)	0.654	1.14 (0.69–1.89)	0.616
Body weight, per 1 kg increment	0.99 (0.97–1.01)	0.542		
BMI, per 1 kg/m^2^ increment	0.98 (0.93–1.04)	0.546		
APACHE II score, per 1-point increment	1.04 (1.00–1.08)	0.052		
APACHE II score ≥ 25	1.08 (0.65–1.81)	0.761		
SOFA score, per 1-point increment	1.05 (0.98–1.12)	0.145	1.02 (0.95–1.09)	0.612
CCI, per 1-point increment	1.07 (0.98–1.17)	0.128		
mNUTRIC score, per 1-point increment	1.19 (1.003–1.41)	0.046 *		
Albumin, per 1 g/dL increment	0.46 (0.29–0.72)	0.001 **	0.49 (0.30–0.78)	0.002 **
Emergency admission	1.05 (0.63–1.75)	0.856		

Univariable and multivariable Cox proportional hazards models for in-hospital mortality. Multivariable Cox proportional hazards regression analysis was performed. Achieving day-7 energy adequacy (≥70% of estimated requirements) was independently associated with reduced mortality risk, and higher albumin levels were also associated with lower in-hospital mortality risk. * *p* < 0.05 and ** *p* < 0.01 indicate statistical significance.

**Table 3:** Baseline and clinical characteristics of the achievement and nonachievement groups.

	Total (*n* = 507)	Calorie Intake/Target Calorie (%) (Day 7)		*p* Value
		<70% (*n* = 152)	≥70% (*n* = 355)	
Age, median (IQR)	69 (57–80)	68 (55–77)	70 (59–80)	0.024 *
Gender, *n* (%)				0.210
Female	208 (41.0%)	56 (36.8%)	152 (42.8%)	
Male	299 (59.0%)	96 (63.2%)	203 (57.2%)	
Body weight (kg), median (IQR)	63.5 (55.2–73.0)	65.0 (58.1–75.0)	62.5 (54.4–72.0)	0.022 *
BMI (kg/m^2^), median (IQR)	24.1 (21.7–27.3)	24.4 (22.1–27.7)	24.1 (21.6–27.2)	0.237
APACHE II score, median (IQR)	26 (21–30)	27 (22–32)	25 (21–30)	0.045 *
APACHE II score ≥ 25, *n* (%)	292 (57.6%)	96 (63.2%)	196 (55.2%)	0.097
SOFA score, median (IQR)	9 (6–12)	10 (7–13)	9 (6–12)	0.009 **
CCI, median (IQR)	4 (2–6)	4.5 (2–7)	4 (2–6)	0.539
mNUTRIC score, median (IQR)	7 (6–8)	7 (6–8)	7 (6–8)	0.194
mNUTRIC score ≥ 5, *n* (%)	457 (90.1%)	140 (92.1%)	317 (89.3%)	0.331
Ventilator day, median (IQR)	11 (8–17)	10 (8–16)	11 (8–17)	0.316
Days of ICU stay, median (IQR)	13 (9–20)	13 (9–20)	13 (10–20)	0.225
Days of hospital stay, median (IQR)	27 (20–39)	26 (19–36)	28 (20–41)	0.107
Emergency admission, *n* (%)	353 (69.6%)	101 (66.4%)	252 (71.0%)	0.309
Lab data, median (IQR)				
Hgb (g/dL)	9.6 (8.3–11.8)	9.3 (7.9–11.6)	9.7 (8.4–11.9)	0.048 *
CRP (mg/dL)	7.3 (1.9–16.7)	9 (2.9–17.9)	6.8 (1.7–16.3)	0.065
Albumin (g/dL)	2.9 (2.5–3.3)	2.7 (2.3–3.1)	2.9 (2.5–3.3)	0.002 **
EN ratio, median (IQR)	92.6 (79.3–97.7)	82.1 (60.3–94.3)	94.7 (86.8–98.5)	<0.001 **
EN only, *n* (%)	40 (7.9%)	5 (3.3%)	35 (9.9%)	0.012 *
IV caloric exposure only, *n* (%)	2 (0.4%)	1 (0.7%)	1 (0.3%)	1.000 *
Day 3, median (IQR)				
Calorie intake (kcal)	1077 (754–1379)	887 (515–1249)	1167 (850–1427)	<0.001 **
Calorie intake/target calorie (%) ≥ 70, *n* (%)	267 (52.7%)	55 (36.2%)	212 (59.7%)	<0.001 **
Calorie intake/target calorie (%)	73.8 (48.5–96.1)	56.1 (35.8–84.4)	79.8 (55.6–99.7)	<0.001 **
Calorie intake (kcal/kg BW)	16.8 (11.1–22.6)	13.1 (8.0–19.6)	18.1 (12.4–23.8)	<0.001 **
Protein intake (g/kg BW)	0.7 (0.4–0.9)	0.5 (0.2–0.8)	0.7 (0.5–1.0)	<0.001 **
Protein intake ≥1 g/kg, *n* (%)	102 (20.1%)	12 (7.9%)	90 (25.4%)	<0.001 **

Demographic factors, illness severity scores, laboratory markers, inflammatory indicators, and early nutritional intake were compared between the groups. Continuous variables were analyzed using the Mann–Whitney U test, and categorical variables were analyzed using the chi-square test or Fisher’s exact test, as appropriate. * *p* < 0.05 and ** *p* < 0.01 indicate statistical significance.

**Table 4:** Predictors of day-7 energy adequacy.

	Univariable Model		Multivariable Model	
	OR (95% CI)	*p* Value	OR (95% CI)	*p* Value
Age, per year increment	1.01 (1.002–1.03)	0.019 *	1.01 (1.00–1.03)	0.038 *
Age ≥ 65	1.32 (0.90–1.94)	0.154		
Male vs. Female	0.78 (0.53–1.15)	0.211	0.90 (0.60–1.36)	0.626
Body weight, per kg increment	0.98 (0.97–0.998)	0.026 *	0.99 (0.98–1.01)	0.192
BMI (kg/m^2^), per 1 kg/m^2^ increment	0.97 (0.93–1.01)	0.114		
APACHE II score, per 1-point increment	0.97 (0.94–0.999)	0.040 *		
APACHE II score ≥ 25	0.72 (0.49–1.06)	0.098		
SOFA score, per 1-point increment	0.93 (0.88–0.98)	0.007 **	0.95 (0.90–1.01)	0.101
CCI, per 1-point increment	0.98 (0.91–1.05)	0.495		
mNUTRIC score, per 1-point increment	0.93 (0.82–1.04)	0.204		
mNUTRIC score ≥ 5	0.72 (0.36–1.41)	0.333		
Albumin, per 1 g/dL increment	1.70 (1.22–2.36)	0.002 **	1.61 (1.14–2.29)	0.007 **
Emergency admission	1.24 (0.82–1.86)	0.309		

Logistic regression analysis of factors associated with achieving ≥70% energy adequacy on ICU day 7. Older age and higher serum albumin levels were independently associated with successful target attainment in the multivariable model. * *p* < 0.05 and ** *p* < 0.01 indicate statistical significance.

## Data Availability

The data presented in this study are available from the corresponding author upon reasonable request. The data are not publicly available because of privacy and ethical restrictions.

## Supplementary Materials

The following supporting information can be downloaded at: https://www.mdpi.com/article/10.3390/nu18101545/s1, Table S1: Predictors of in-hospital mortality after excluding patients who died on ICU day 7; Table S2: Predictors of in-hospital mortality after excluding patients who died within the first 8 ICU days; Table S3: Predictors of in-hospital mortality after excluding patients who died within the first 9 ICU days; Table S4: Route-adjusted Cox model for in-hospital mortality; Figure S1: Sensitivity Kaplan–Meier survival plot indicating the ICU day-9 landmark according to day-7 energy adequacy.

## Abbreviations

The following abbreviations are used in this manuscript:

APACHE II	Acute Physiology and Chronic Health Evaluation II
BMI	Body Mass Index
CCI	Charlson Comorbidity Index
CRP	C-Reactive Protein
ESPEN	European Society for Clinical Nutrition and Metabolism
EN	Enteral Nutrition
ICU	Intensive Care Unit
IQR	Interquartile Range
IV	Intravenous
mNUTRIC	Modified Nutrition Risk in the Critically Ill
SOFA	Sequential Organ Failure Assessment
