# Supplementary material for: Timing of Achieving 70% of Energy Requirements in Critically Ill Patients: Association with In-Hospital Mortality and Predictors in a Real-World Medical ICU
Source: Nutrients. 2026 May 13;18(10):1545. doi: 10.3390/nu18101545 (PMC13210351; doi:10.3390/nu18101545)
Supplement: Supplementary file 1 [file nutrients-18-01545-s001.zip › nutrients-4300986-supplementary.pdf]

Table S1. Predictors of in-hospital mortality after excluding patients who died on ICU day 7.

	Univariable model		Multivariable model	
	HR (95% CI)	*p* value	HR (95% CI)	*p* value
Calories intake/target calorie ≥70 (%)				
Day 3	0.68 (0.41 - 1.12)	0.132		
Day 7	0.56 (0.33 - 0.93)	0.024*	0.56 (0.33 - 0.95)	0.031*
Age, per year increment	1.02 (1.00 - 1.04)	0.071	1.02 (1.002 - 1.04)	0.030*
Male vs Female	1.11 (0.66 - 1.86)	0.690	1.13 (0.67 - 1.9)	0.641
Body weight, per 1 kg increment	0.99 (0.97 - 1.01)	0.493		
BMI, per 1 kg/m^2 increment	0.98 (0.92 - 1.04)	0.502		
APACHE II score, per 1 score increment	1.03 (0.99 - 1.07)	0.146		
APACHE II score ≥ 25	0.99 (0.59 - 1.67)	0.979		
SOFA score, per 1 score increment	1.04 (0.97 - 1.12)	0.227	1.01 (0.94 - 1.09)	0.773
CCI, per 1 score increment	1.07 (0.98 - 1.18)	0.127		
mNUTRIC score, per 1 score increment	1.16 (0.97 - 1.38)	0.098		
Albumin, per 1 g/dL increment	0.45 (0.28 - 0.71)	0.001**	0.47 (0.29 - 0.75)	0.002**
Emergency admission	0.99 (0.59 - 1.66)	0.955		

Univariable and multivariable Cox proportional hazards models for in-hospital mortality. Multivariable Cox proportional hazards regression analysis was performed. Achieving day 7 energy adequacy (≥70% of estimated requirements) was independently associated with reduced mortality risk, and higher albumin levels were also associated with lower in-hospital mortality risk. *$p < 0.05$ and **$p < 0.01$ indicate statistical significance.

Table S2. Predictors of in-hospital mortality after excluding patients who died within the first 8 ICU days.

	Univariable model		Multivariable model	
	HR (95% CI)	*p* value	HR (95% CI)	*p* value
Calories intake/target calorie ≥70 (%)				
Day 3	0.64 (0.38 - 1.09)	0.099		
Day 7	0.55 (0.32 - 0.92)	0.023*	0.55 (0.32 - 0.94)	0.030*
Age, per year increment	1.02 (1.00 - 1.04)	0.087	1.02 (1.001 - 1.04)	0.037*
Male vs Female	1.02 (0.61 - 1.72)	0.936	1.04 (0.61 - 1.76)	0.883
Body weight, per 1 kg increment	0.99 (0.97 - 1.01)	0.587		
BMI, per 1 kg/m^2 increment	0.98 (0.93 - 1.05)	0.617		
APACHE II score, per 1 score increment	1.03 (0.99 - 1.08)	0.128		
APACHE II score ≥ 25	0.97 (0.57 - 1.66)	0.918		
SOFA score, per 1 score increment	1.05 (0.98 - 1.12)	0.205	1.02 (0.94 - 1.10)	0.663
CCI, per 1 score increment	1.08 (0.98 - 1.19)	0.104		
mNUTRIC score, per 1 score increment	1.15 (0.96 - 1.37)	0.123		
Albumin, per 1 g/dL increment	0.46 (0.29 - 0.74)	0.001**	0.48 (0.29 - 0.79)	0.003**
Emergency admission	0.92 (0.54 - 1.56)	0.760		

Univariable and multivariable Cox proportional hazards models for in-hospital mortality. Multivariable Cox proportional hazards regression analysis was performed. Achieving day 7 energy adequacy (≥70% of estimated requirements) was independently associated with reduced mortality risk. Higher serum albumin levels were also associated with lower in-hospital mortality risk. *$p < 0.05$ and **$p < 0.01$ indicate statistical significance.

Table S3. Predictors of in-hospital mortality after excluding patients who died within the first 9 ICU days.

	Univariable model		Multivariable model	
	HR (95% CI)	*p* value	HR (95% CI)	*p* value
Calories intake/target calorie ≥70 (%)				
Day 3	0.66 (0.38 - 1.12)	0.123		
Day 7	0.58 (0.34 - 0.99)	0.045*	0.59 (0.34 - 1.03)	0.061
Age, per year increment	1.02 (1.00 - 1.03)	0.118	1.02 (1.00 - 1.04)	0.056
Male vs Female	0.93 (0.55 - 1.59)	0.796	0.95 (0.56 - 1.63)	0.858
Body weight, per 1 kg increment	0.99 (0.97 - 1.01)	0.459		
BMI, per 1 kg/m^2 increment	0.98 (0.92 - 1.04)	0.496		
APACHE II score, per 1 score increment	1.03 (0.99 - 1.07)	0.204		
APACHE II score ≥ 25	0.95 (0.55 - 1.64)	0.855		
SOFA score, per 1 score increment	1.04 (0.97 - 1.12)	0.284	1.01 (0.94 - 1.09)	0.767
CCI, per 1 score increment	1.07 (0.97 - 1.18)	0.152		
mNUTRIC score, per 1 score increment	1.14 (0.95 - 1.37)	0.155		
Albumin, per 1 g/dL increment	0.45 (0.28 - 0.73)	0.001*	0.46 (0.28 - 0.76)	0.003**
Emergency admission	0.85 (0.50 - 1.47)	0.569		

Univariable and multivariable Cox proportional hazards models for in-hospital mortality. Multivariable Cox proportional hazards regression analysis was performed. Achieving day 7 energy adequacy showed a directionally similar association with lower in-hospital mortality, although the association did not reach statistical significance after multivariable adjustment. Higher serum albumin levels were also associated with lower in-hospital mortality risk. *$p < 0.05$ and **$p < 0.01$ indicate statistical significance.

Table S4. Route-adjusted Cox model for in-hospital mortality

	Univariable model		Multivariable model	
	HR (95%CI)	*p* value	HR (95%CI)	*p* value
Calories intake/target calorie≥70 (%)				
Day 3	0.71 (0.43 - 1.16)	0.171		
Day 7	0.50 (0.30 - 0.82)	0.006**	0.53 (0.32 - 0.89)	0.016*
EN ratio, per 1 percentage increment	0.99 (0.98 - 0.999)	0.036*	0.99 (0.98 - 1.01)	0.322
Age, per year increment	1.02 (1.00 - 1.03)	0.073	1.02 (1.004 - 1.04)	0.018*
Male vs Female	1.12 (0.68 - 1.85)	0.654	1.12 (0.67 - 1.86)	0.666
Body weight, per 1 kg increment	0.99 (0.97 - 1.01)	0.542		
BMI, per 1 kg/m2 increment	0.98 (0.93 - 1.04)	0.546		
APACHE II score, per 1 score increment	1.04 (1.00 - 1.08)	0.052		
APACHE II score ≥ 25	1.08 (0.65 - 1.81)	0.761		

SOFA score, per 1 score increment	1.05 (0.98 - 1.12)	0.145	1.01 (0.94 - 1.09)	0.724
CCI, per 1 score increment	1.07 (0.98 - 1.17)	0.128		
mNUTRIC score, per 1 score increment	1.19 (1.003 - 1.41)	0.046*		
Albumin, per 1 g/dL increment	0.46 (0.29 - 0.72)	0.001**	0.51 (0.32 - 0.82)	0.006**
Emergency admission	1.05 (0.63 - 1.75)	0.856		

Univariable and multivariable Cox proportional hazards models for in-hospital mortality. Multivariable Cox proportional hazards regression analysis was performed. Achieving day 7 energy adequacy (≥70% of estimated requirements) was independently associated with reduced mortality risk. Higher serum albumin levels were also associated with lower in-hospital mortality risk. *$p < 0.05$ and **$p < 0.01$ indicate statistical significance.

Figure S1. Sensitivity Kaplan–Meier survival plot indicating the ICU day-9 landmark according to day-7 energy adequacy.

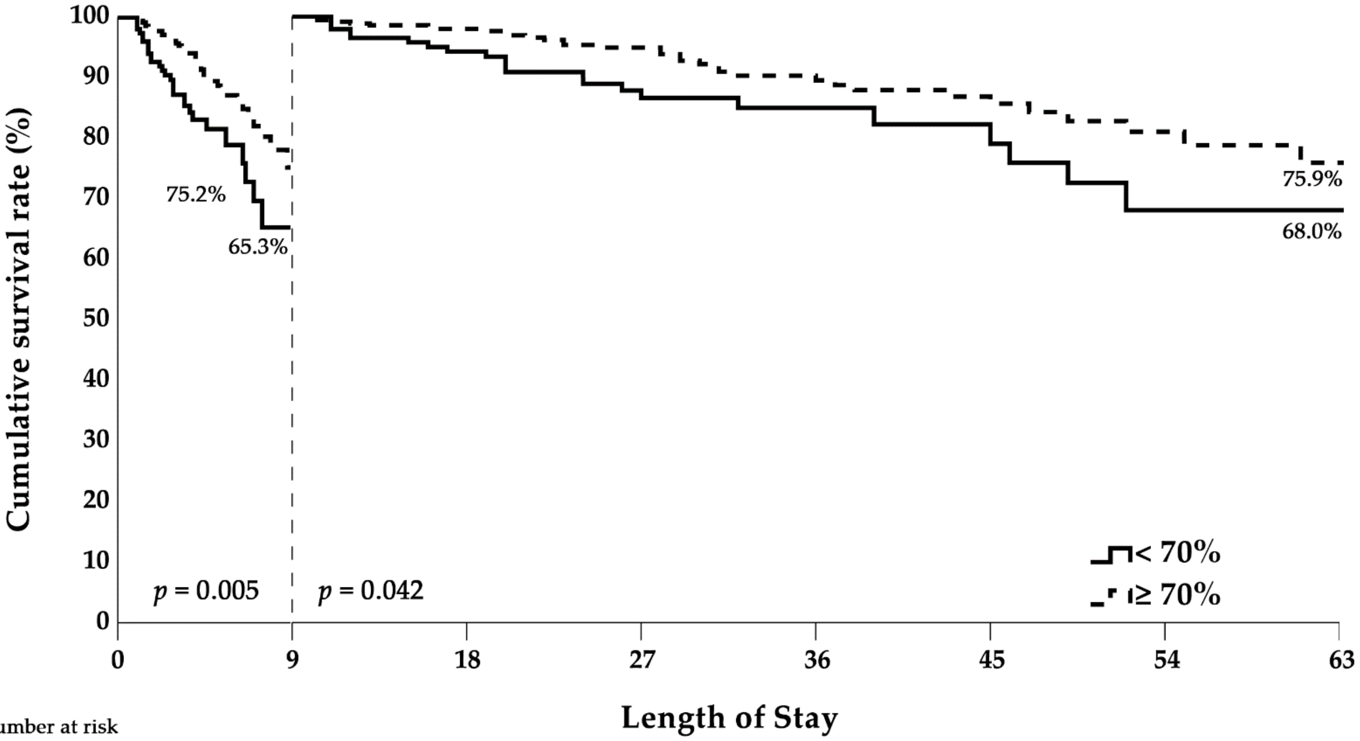

Number at risk

< 70%	152	142	117	68	37	25	13	10
≥ 70%	355	351	288	180	113	66	37	23

The Kaplan–Meier survival curves are shown according to achievement of ≥70% of estimated energy requirements on ICU day 7. The ICU day-9 landmark is indicated in the figure. The post-landmark comparison was performed among patients who remained at risk after excluding those who died within the first 9 ICU days. The portion of the curve before ICU day 9 is displayed only for visual context and was not used as the basis of the post-landmark sensitivity comparison. Statistical comparison after the ICU day-9 landmark was performed using the log-rank test. $p < 0.05$ indicated statistical significance.
